# Supplementary material for: Cannabis stigma and symptom management considerations in cancer survivors: a mixed-methods exploration of patient perspectives
Source: Support Care Cancer. 2026 Mar 13;34(4):313. doi: 10.1007/s00520-026-10523-2 (PMC12982309; doi:10.1007/s00520-026-10523-2)
Supplement: Supplementary file 1 — Supplementary Material 1 (DOCX 18.8 KB) [file 520_2026_10523_MOESM1_ESM.docx]

Codebook for Project:

**Cannabis Stigma and Symptom Management Considerations in Cancer Survivors: A Mixed-Methods Exploration of Patient Perspectives**

Theme: Cancer as a Unique Condition: Special Treatment and Exemptions

**Codes:**

- **“cancer as an acceptable reason”**: when patients indicate getting excused by friends or family or society for their use of cannabis due to cancer diagnosis
- **“cancer care”**: reference about the specialized care you receive at a cancer hospital as compared to noncancer care

Theme: Cancer Experience: Identity, Judgement, and Inequities

**Codes:**

- **“perfect patient”**: when patients explain a tendency to be a "perfect" patient, e.g. not being "annoying" with needing more attention, or help, or having problems, etc.
- **“judgement from other survivors”**: when a patient speaks about feeling judgement from other cancer survivors
- **“not being believed”**: patient describing that they were not being believed about their pain
- **“racial disparities”**: mention of differing treatment, prescriptions, etc. due to racial difference**s**
- **“women social norms and cancer”**: discussion about being a woman and a patient in the cancer healthcare system

Theme: Healthcare Provider Interaction: Attitudes and Reactions to Cannabis Use

**Codes**:

- **“conversation with doctor – negative”**: when a patient references a conversation with their provider about cannabis that suggested a negative experience
- **“conversation with doctor – neutral”**: discussion about a conversation with their doctor that doesn't appear to be perceived and positive or negative, more-so a neutral, matter of fact tone
- **“conversation with doctor – positive”**: when a patient discusses a positive experience discussing cannabis with their provider
- **“harm reduction conversation with provider”**: when a patient discussed a conversation with their provider where the provider has a harm reduction approach - not telling patients to stop using, or that they shouldn't be using; rather, suggesting safer methods to use.
- **“judgement from provider”**: when a patient makes a reference to feeling judged by their healthcare provider
- **“perception of how doctors view cannabis”**: when a patient makes mention of how they perceive their providers to view cannabis
- **“provider initiating conversation about cannabis”**: reference to a patient’s provider suggesting cannabis
- **“reaction to conversation with doctor”**: when a patient discusses their reaction to the conversation with healthcare provider

Theme: Navigating Medicinal Cannabis Use: Concerns and Considerations

- **“cannabis dependence’**: references feeling dependent on cannabis and/or increased tolerance to use (e.g., can't cut down or stop using; need to use more of cannabis for same effect)
- **“comparing cannabis to other recreational substances”**: when patients make some reference to using cannabis in the same way others use recreational drugs
- **“concern with children's perspectives”:** when a patient describes some concern with their children, or people they care for, knowing that they use cannabis
- **“difference between recreational vs medical”**: when patients compare medical use versus recreational use
- **“influence of C4C study [parent study]”**: when a patient makes reference about how being in the C4C study influenced a decision
- **“insurance and cannabis”**: when a patient refers to insurance issues and how this may legitimize cannabis or help with costs
- **“judgement from friends or family”**: when a patient speaks about either the presence or absence of judgement from friends or family
- **“legality issues”**: mention of problems or consequences of cannabis being illegal (either federally or in certain places)
- **“negative effects of cannabis”**: references adverse effects or negative side effects of cannabis (e.g., difficulty concentrating; inability to drive, etc.)
- **“othering”**: when a patient makes a comment that seems to separate themselves from other people who use cannabis
- **“pain needing to be ‘bad enough’ to use cannabis”**: when a patient describes that pain would need to be at a certain threshold to consider using cannabis
- **“questioning use”**: when a patient describes a time where they question their use for any reason
- **“views on the general climate on cannabis use”**: when a patient makes reference of their subjective view of cannabis in society, or medicine, broadly. Positive, negative, or neutral

Theme: Navigating Opioid Use: Pain, Stigma, and Fears of Addiction

- **“labeling - opioid use”**: use of stigmatizing labels as it relates to opioids use
- **“opioid stigma”**: mention of word stigma related to opioids
- **“subjective view of opioids”**: when a patient describes their opinion of an opioid

Theme: Patient Empowerment and Self-Directed Pain Management

- **“empowerment”**: when patients feel empowered to manage their pain however, for whatever reasons
- **“going against medical advice”**: when a patient makes reference to not using a substance as advised
- **“rejecting judgement”**: rejecting what others might thing about cannabis use
- **“taking matters into their own hands”**: when a patient mentions something about needing to try non-traditional or non-Western medications due to limited options available or hope in the systems

Theme: Perceived Stigma and/or the Concealment of Cannabis Use

- **“access issues – opioids”**: when a patient describes some difficulty in accessing opioids due to their use of cannabis (e.g., drug tests, medical marijuana cards, etc.)
- **“cannabis nondisclosure”**: when a patient expresses their choice to not disclose their cannabis use to their healthcare provider
- **“fear of judgement”**: when a patient makes note of the fear of judgement being part of the decision to use cannabis
- **“fear of oversight”**: when a patient makes reference to fearing oversight from the government, or medical institution, or other governing bodies who may be able to see they are registered for cannabis, using, etc.
- **“feeling shame/guilt”**: when a patient discusses feelings of shame or guilt about their use
- **“labeling – cannabis” **:** when the patient uses a label that stigmatizes cannabis use (i.e., druggie, addict, pothead, etc.)
- **“negative stereotyping” ****: when a patient refers to a stereotype that might relate to cannabis use, such as faking symptoms to get drugs, using other drugs, selling drugs, etc.
- **“power asymmetry” ****: reference to losing access to medications through the healthcare system using cannabis

Theme: Weighing Cannabis Against Opioid Risks and Concerns

- **“cannabis as alternative to opioids”**: when a patient describes using cannabis as an alternative to opioids, including that it's "safer"
- **“fear of opioid addiction”**: when a patient expresses concern about becoming addicted to opioids

Theme: When and Why Patients Choose Cannabis for Symptom Relief

- **“cannabis as distraction”**: citing use of cannabis for reasons other than pain, here, for distraction
- **“considering use”**: when a patient mentions consideration of cannabis use
- **“nonuser view of cannabis”**: any mention of how a nonuser views the use of cannabis
- **“reason for nonuse”**: when a patient mentions their reason for not using
- **“reason for use”**: when a patient notes the reason they use it - what benefits they're getting.

** Codes marked with two asterisks were defined a priori based on the stigma framework.
