# Supplementary material for: Cannabis stigma and symptom management considerations in cancer survivors: a mixed-methods exploration of patient perspectives
Source: Support Care Cancer. 2026 Mar 13;34(4):313. doi: 10.1007/s00520-026-10523-2 (PMC12982309; doi:10.1007/s00520-026-10523-2)
Supplement: Supplementary file 2 — Supplementary Material 2 (DOCX 21.3 KB) [file 520_2026_10523_MOESM2_ESM.docx]

Appendix A

Introduction

Hi all! Thank you so much for coming in today. My name is Sera and I’ll be asking the group some questions in hopes that it can start a conversation about how people handle symptoms like pain, sleep, nausea, etc.

So, we know that many people choose to use cannabis to help manage some cancer- and cancer-treatment related symptoms, while many do not. We’re interested in learning about what helped you make the decision to add cannabis into your treatment regimen—or not. Additionally, we know that people have had varying experiences with discussing cannabis use with their providers, so we’re interested in learning about ways in which your providers have been helpful or unhelpful during these talks.

*They complete surveys & consent. Allow anyone not willing to be recorded or participate to leave the Zoom call.*

****START RECORDING****

Ok, great! Now we can move into the discussion portion of this session. Before we get started, we want everyone to feel safe sharing during the group. We ask that everyone please respect the privacy of others and do not repeat what is discussed in this focus group. We have a lot of questions to go through, and we want to make sure that everyone has an opportunity to speak, so I may need to cut you off at some points for the sake of time, so I apologize in advance! Are there any questions before we begin?

*Answer questions as needed.*

Great! Let’s start with introductions.

Thanks everyone!

**Cannabis users**

1. What are your general feelings about cannabis use for symptom management? How do you think this differs or aligns with general society’s feelings about medicinal cannabis use?
2. What are your general feelings about opioid use for symptom management? How do you think this differs or aligns with general society’s feelings about opioid use?
3. What was your decision-making process like when deciding if you wanted to use cannabis? (*follow-up prompts if necessary: what types of things did you hear about cannabis use from friends/families/news before you decided to try it? Did these conversations have an impact on your decision?*)
4. How do you feel about traditional symptom management tools like opioids? (*follow-up prompts: Did this impact your decision to use cannabis? did you have a personal experience with opioids? Did conversations about opioids in the news or society in general impact any of these feelings?*)
5. What was it like to discuss your cannabis use with your oncology provider(s)? Doctors, PAs, nurses, etc. (*follow-up prompts if they had a discussion with provider: was this something you were comfortable disclosing from the beginning, or did it take some time to feel comfortable? Did their responses make you feel any certain emotions, or have an effect on future conversations? For example, did you find yourself less comfortable to talk about your use with providers?*

*Follow-up prompts if no discussion with provider: what kept you from asking your provider about cannabis?*)

1. What experiences have led you to question your cannabis use, if any, and why? (*follow-up prompt: did discussions about cannabis or reactions make you feel uncertain about if you should continue using cannabis)*
2. How did you perceive your care to change from providers after disclosing your use, if at all? (*follow-up prompt: can you tell me more about that? Or if needed: did doctors ask you more questions, show you more concern, limit other medication prescriptions, etc.?)*
3. What kinds of questions arose about other substance use or your daily lifestyle following your disclosure to healthcare providers, if any? (*follow-up prompt: was substance use a topic that you and your provider(s) ever discussed before)*
4. Can you share any instances where someone's reaction to your use influenced your decision to continue or stop using cannabis? (*follow-up prompt: did you ever try to use less or take a break?*)
5. Do you feel like individuals with cancer are subject to judgement for how they choose to manage their symptoms? If yes, in what ways? (*follow-up prompts: can you tell me more about that? Do people make comments about what is “right” or “wrong” in terms of how you manage your symptoms?)*
6. Do you feel like people with other conditions are judged for using cannabis to help manage their symptoms? For example, people with chronic pain or people who use it to manage anxiety.
7. We know that having a cancer diagnosis often involves attending many different types of appointments, how have your treatments (i.e., medication and/or cannabis side effects) affected, if at all, your ability to attend appointments? (*for example, using opioids or cannabis and managing side effects while needing to make appointments)*
8. How successful is your current treatment regimen in helping you manage symptoms? (*follow-up prompt: If not very, do you wonder about alternative options? What is stopping you from trying other treatments?)*
9. Are there any other discussion points that I missed and should have asked?

**Cannabis nonusers**

1. What are your general feelings about cannabis use for symptom management? How do you think this differs or aligns with general society’s feelings about medicinal cannabis use?
2. What are your general feelings about opioid use for symptom management? How do you think this differs or aligns with general society’s feelings about opioid use?
3. Who has considered using cannabis to help manage their symptoms?
   1. If yes: what factors influenced your decision to not use cannabis to help manage your symptoms? (*follow-up prompts: what types of things did you hear about cannabis use from friends/families/news? Did these conversations have an impact on if you considered it or not?)*
   2. If no: do you know anyone who has ever used cannabis? If yes, how did that play a role in your decision to not consider use of cannabis?
      1. Follow-up questions: why do you think you’ve never considered it?
4. For people who have used cannabis for symptom management before, please share about your experience and how it has influenced your decision to not use this substance to manage symptoms now. (*follow-up prompt: can you tell us more about the reason(s) you stopped?)*
5. How do you feel about traditional symptom management tools like opioids? (*follow-up prompts: did you have a personal experience with opioids? Did conversations about opioids in the news or society in general impact any of these feelings?*)
   1. Dependence?
6. If you use opioids, how have healthcare providers, family members, or friends responded to your opioid use, and how did those reactions make you feel?
7. We know that having a cancer diagnosis often involves attending many different types of appointments, how have your treatments (i.e., medication and/or cannabis side effects) affected, if at all, your ability to attend appointments? (*for example, using opioids and managing side effects while needing to make appointments)*
8. Do any of you feel like individuals with cancer are subject to judgement for how they choose to manage their symptoms? If yes, in what ways? (*follow-up prompts: can you tell me more about that? Do people make comments about what is “right” or “wrong” in terms of how you manage your symptoms?)*
9. How successful is your current treatment regimen in helping you manage symptoms? (*follow-up prompt: If not very, do you wonder about alternative options? What is stopping you from trying other treatments?)*
10. Are there any other discussion points that I missed and should have asked?
